# Aging Weakens Memory for Schema-Deviant Objects and Decouples Gaze Sampling from Retrieval Decisions

**DOI:** 10.3390/brainsci16030289

**Published:** 2026-03-05

**Authors:** Hong-Zhou Xu, Sheng-Yin Huan

**Affiliations:** Faculty of Psychology, Southwest University, Chongqing 400715, China

**Keywords:** aging, schemas, false memory, eye tracking, representational similarity analysis

## Abstract

**Background/Objectives:** Schemas support memory, but it is unclear how aging affects remembering when events deviate from schemas in different ways. We tested whether age differences depend on the integrability of schema-deviant object configurations and whether eye movement evidence sampling tracks retrieval decisions. **Methods:** Young and older adults completed a Restructured Object Memory Task. They encoded objects that were non-restructured (schema-consistent), reasonably restructured (deviant but integrable), or unreasonably restructured (deviant and non-integrable). At retrieval, participants made three-alternative forced-choice judgments while eye movements were recorded. Subjective ratings assessed perceived deviation, and representational similarity analysis related eye-movement patterns to memory confusions. **Results:** Older adults showed poorer cue discrimination and object memory, with the largest deficits for restructured objects. Their errors were schema-consistent, often selecting the typical object when targets were restructured. Ratings confirmed the intended deviation ordering, but older adults differentiated conditions less. Eye movements showed that young adults showed the highest target viewing proportion for reasonably restructured targets, and longer fixation durations for unreasonably restructured targets. In young adults, eye-movement representational structure tracked memory confusions. In older adults, early orienting and fixation duration were less predictive of choices, consistent with weaker coupling between sampling and decision. **Conclusions:** Aging was associated with poorer memory for schema-deviant objects, consistent with reduced representational fidelity and reduced flexibility in how online visual evidence is sampled and used when prior knowledge conflicts with new configurations.

## 1. Introduction

Human memory is not a literal record of the past. Rather, the process of remembering is constructive. It uses stored knowledge to interpret experiences, fill in missing details, and create coherent representations of events [1,2,3]. A central mechanism that fosters this constructive capacity is schemas: organized frameworks of knowledge that help predict incoming input by organizing regularities in the environment [4,5]. Schemas provide an interpretive framework that supports the rapid encoding and retrieval of information when expectations for new experiences are met. In situations where information is lacking, schemas often enhance understanding and memory [6,7,8]. However, schema-driven reconstruction may also introduce systematic distortions. Memory that is informed but partially detailed may favor schema-confirmatory inferences, resulting in confident but inaccurate recall [9,10,11,12].

Maintaining a balance between schematic benefits and distortion costs may be especially important in healthy aging. At the same time, older adults tend to rely more on schematic or gist-like information, which can be adaptive but also increases the risk of schema-consistent false memory [13,14,15,16,17]. Importantly, schema violation is not a single psychological category: some deviations are surprising yet conceptually integrable, whereas others are so implausible that they resist integration. Distinguishing these forms of deviation can clarify when schemas support remembering and when they promote distortion later in life.

One prominent account of these age-related changes emphasizes cognitive control. Successful encoding and retrieval of novel, schema-violating information requires significant top-down control to override automatic schematic associations and allocate resources to process unique perceptual and conceptual elements [18,19,20,21]. Prominent theories of cognitive aging, such as the inhibitory deficit hypothesis and the frontal aging hypothesis, posit that a primary driver of age-related cognitive decline is a reduction in the efficacy of executive functions subserved by the prefrontal cortex [22,23,24,25]. This decline in control may impede older adults’ capacity to suppress irrelevant, schema-driven thoughts and to engage in the elaborative processing necessary to establish a durable memory trace for unexpected information [24,26,27,28,29]. While behavioral measures provide a valuable snapshot of memory’s final output, they offer limited insight into the real-time cognitive dynamics of retrieval. In contrast, eye tracking provides a powerful, high-resolution lens into these processes because gaze patterns reflect the competition between memory representations and the allocation of attention during decision-making [30,31,32]. Thus, eye-movement measures can complement accuracy-based outcomes by showing how memory-guided selection unfolds in real time.

To address these gaps, we examined age-related differences in memory for objects that changed in relation to everyday schemas using a Restructured Object Memory Task (ROMT). During each trial, participants first viewed a partial image of an object as a cue. They were then asked to mentally complete the image before the full object was revealed. This created a conflict between participants’ cue-driven expectations (i.e., typical schemas) and the object’s actual configuration. Participants encountered three types of objects: non-restructured objects that conformed to typical schemas at both encoding and retrieval; reasonably restructured objects that violated schemas but remained conceptually integrable (i.e., the cue–object combination supports a coherent contextual/functional/thematic explanation that preserves core category constraints); and unreasonably restructured objects that were schema-deviant and non-integrable (i.e., the modification impedes such coherent explanation and/or violates core category constraints). Here, integrability refers to the ease with which a coherent explanation can be generated linking the cue to the whole object, allowing accommodation into the activated schema; importantly, this is separable from the overall degree of deviation. This RR–UR distinction follows the existing theoretical accounts summarized above, which emphasize that schema deviations can differ in how readily they can be reconciled with prior knowledge versus remaining difficult to integrate and thus relying more on item-specific representations [18,19,20,21,33]. Against this backdrop, we expected age-related memory costs to scale with integrability: older adults should show relatively smaller costs for integrable RR deviations but larger costs for non-integrable UR deviations, whereas young adults should show comparatively limited differentiation across deviation types. This design allowed us to test whether age-related vulnerabilities are comparable across degrees of schema deviation or if they are amplified when successful remembering requires stronger resolution of the conflict between prior knowledge and incoming information.

Our data revealed a pronounced dissociation by age group. The memory of young adults was intact for all object types, whereas the memory of older adults was significantly impaired for both reasonably and unreasonably restructured objects. Critically, older adults’ performance followed a graded pattern consistent with the integrability manipulation (NR > RR > UR), indicating that age-related vulnerability was amplified when deviations were less readily reconciled with prior knowledge. Older adults showed a pronounced bias toward schema-consistent false memories. They frequently misclassified restructured objects as their non-restructured counterparts. Such misclassifications were comparatively rare in young adults. This pattern aligns with the broader perspective that schema-driven false alarms and misattributions (errors of commission rather than omission) provide valuable insights into age-related changes in memory, surpassing what can be inferred from overall accuracy or omission-based measures alone [17,34].

The eye movement results also clarified the mechanisms by which this discrepancy occurred. Young adults adapted their visual inspection during retrieval, spending more time fixating on more extremely schema-deviant targets. This pattern suggests more sustained evidence evaluation when the target most strongly conflicted with cue-activated schemas, increasing the need to discriminate it from schema-consistent lures. In contrast, older adults demonstrated reduced modulation of processing time in response to object type, indicating less flexible allocation of attention to schema deviation. Overall, representational similarity analysis yielded convergent evidence that memory decisions were more closely related to gaze-based sampling in young adulthood than in old age. Specifically, the association between the representational structure of eye movements and memory choices was stronger in young adults for early orienting and fixation duration. This indicates greater integration between online sampling and the information underlying object memory. Overall, these findings suggest that older adults exhibit less gaze-based modulation as schema deviation increases and reduced alignment between gaze sampling patterns and subsequent memory selections. These patterns are consistent with age-related differences in conflict-sensitive control and/or reduced item-specific mnemonic specificity (i.e., lower representational fidelity in distinguishing restructured targets from schema-consistent lures), while also allowing for alternative explanations (e.g., perceptual or salience-related discrepancies).

## 2. Materials and Methods

### 2.1. Participants

Prior to the experiment, G*Power 3.1 (http://www.gpower.hhu.de/; RRID: SCR_013726) was used to calculate the required sample size based on repeated measures ANOVA (within–between interaction) targeting the age × condition interaction on true object memory across NR, RR, and UR conditions with the parameters *f* = 0.25, *α* = 0.05, and 1 − *β* = 0.95, indicating a required sample size of 44 participants. To account for potential attrition, the study recruited 60 adults with no history of psychiatric or neurological disorders, including 30 young participants from Southwest University and 30 community-dwelling older adults. The older adults were required to have a Montreal Cognitive Assessment (MoCA) score of ≥22 [35,36]. One young participant was excluded because their overall true object memory accuracy, computed as the mean accuracy averaged across NR, RR, and UR, was more than three standard deviations below the sample mean (overall accuracy = 0.35). Additionally, three young participants and three older participants were excluded because the number of trials with gaze data meeting quality control criteria in any condition fell below the 30% threshold. The final sample consisted of 55 valid participants: 26 young adults (age range 18–26 years, *M* = 21.00, *SD* = 1.79; male/female = 6/20) and 29 older adults (age range 59–75 years, *M* = 67.69, *SD* = 4.14; male/female = 7/22). In the final older-adult sample included in all analyses (N = 29), MoCA scores ranged from 22 to 29 (*M* = 25.97, *SD* = 1.78).

This study was approved by the Ethics Committee of the Department of Psychology at Southwest University (Approval No.: H23122) and conducted in accordance with the principles of the Declaration of Helsinki. All participants provided written informed consent and received compensation for their involvement.

### 2.2. Experimental Materials

**Figure 1:**
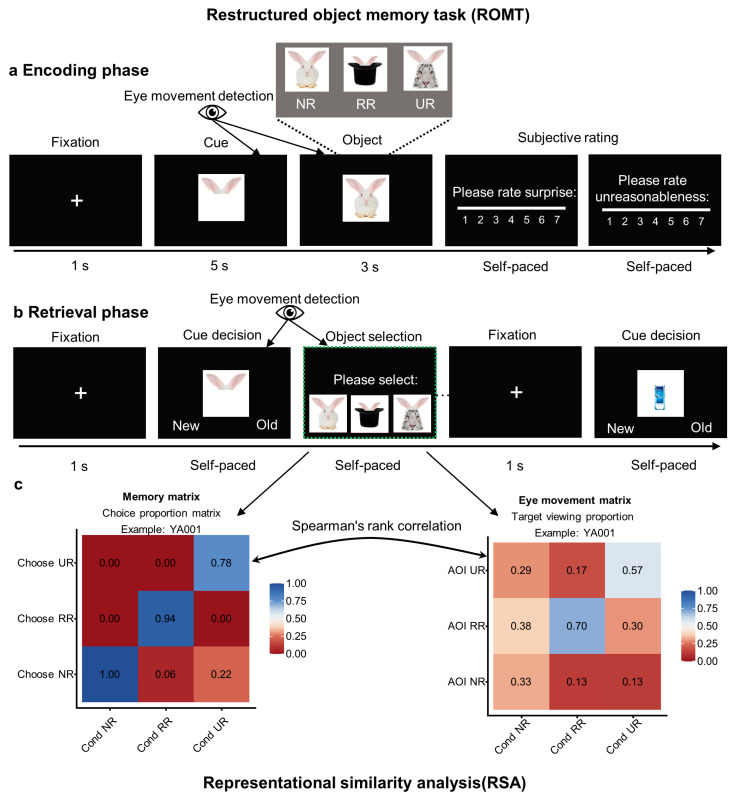
The experimental design and procedure. (**a**) The encoding phase and three object conditions are exemplified. (**b**) The retrieval phase. The object selection screen in the dotted box is only displayed if there is an “old” response to an old cue that had been presented in the encoding phase. (**c**) The computational process of representational similarity analysis (taking YA001 as an example). YA, young adults; NR, non-restructured; RR, reasonably restructured; UR, unreasonably restructured; AOI, area of interest.

The present study created three types of objects that differ in schematic deviation and integrability. The stimulus set consists of 60 groups, with each group containing three object variants: non-restructured (NR), reasonably restructured (RR), and unreasonably restructured (UR). NR objects are unmodified, typical objects (e.g., a rabbit). RR objects are reasonably cross-object restructured objects that deviate from the typical schema while maintaining integrability (e.g., a magician’s hat with rabbit ears). UR objects significantly deviate from the schema and lack integrability (e.g., a tiger with rabbit ears; see Figure 1a). The stimuli were generated based on rule-based constraints. RR objects were selected to provide easily interpretable relationships, such as reasonable context, functional, or thematic connections. UR objects were designed to minimize interpretability and prevent coherent functional explanations from forming. Drawing on established principles of conceptual combination [37,38,39], we treated a restructured object as more “integrable” when (a) a coherent relation between the cue and the whole object could be readily inferred (e.g., contextual/functional/thematic), and (b) the resulting combination preserved category coherence by remaining compatible with core constraints of the base concept, yielding a single interpretable representation without requiring ad hoc assumptions. Conversely, we treated objects as less “integrable” when the modification substantially disrupted category coherence and did not naturally afford a coherent explanatory relation, consistent with the goal of minimizing interpretability for UR objects. To reduce low-level perceptual confusion, each stimulus group shared the same typical cues and key visual components (e.g., an identical “rabbit ears” cue in the NR, RR, and UR triads), ensuring that the cues were schema-consistent and that the features were typical. Meanwhile, schematic deviation was manipulated at the level of the complete object. Thus, NR, RR, and UR differ in how the complete object matches (or violates) the schema evoked by the cue and in how easily the restructured object can be integrated into existing knowledge. Conceptually, this operationalization aligns with theories distinguishing schema violations that can be accommodated via links to prior knowledge from violations that resist such accommodation and are more likely to depend on item-specific representations [18,19,20,21,33]. To ensure that RR and UR objects reflect systematic differences in reasonableness and integrability rather than arbitrary selection, established principles of conceptual combination were referenced [37,38,39], and the expected ranking was validated through participant ratings of surprise and unreasonableness. Conceptually, surprise reflects the actual object presented relative to cue-activated schemas, whereas unreasonableness more directly indexes perceived plausibility/coherence of the resulting configuration. Importantly, these ratings were used to validate the intended subjective ordering across NR/RR/UR, not to define the categories post hoc.

### 2.3. Design and Procedures

This experiment used a Restructured Object Memory Task (ROMT), which was developed by the research group. The ROMT has two phases: encoding and retrieval. To eliminate the influence of time pressure on the participants’ encoding and retrieval processes, all response interfaces were designed with a self-paced, untimed mechanism. Before beginning the task, participants were instructed to pay attention to and remember both the cues (partial images) and the subsequent presentation of the complete objects, as both would be tested in the retrieval phase. During the encoding phase (Figure 1a), each trial began with a one-second fixation point, followed by a five-second presentation of a cue (e.g., rabbit ears) to activate schema-based holistic associations. Then, a complete object was presented for three seconds for learning and encoding. Because the presented objects may differ from the participants’ associative outcomes, inconsistencies in object morphology may occur, even under the NR condition. Thus, conflicts could arise between activated schemas and actual objects under the NR, RR, and UR conditions. To enhance engagement, participants were required to rate their surprise and unreasonableness on a 1–7 Likert scale after each object was presented. This phase comprised a total of 60 trials (20 per condition), with each cue uniquely paired with a complete object only once. The experimental materials were balanced using a Latin square design. The retrieval test commenced immediately after the encoding phase.

During the retrieval phase (Figure 1b), participants first see a cue and must judge whether it is “old” or “new.” For an “old” response, they must perform a two-stage response and select the corresponding complete object from a display of three options. This display always includes the previously learned target object and two distractors from different categories but from the same cue. In RR trials, for instance, the distractors consist of one schema-consistent distractor (i.e., an NR object from the same group) and one schema-inconsistent distractor (i.e., a UR object from the same group). In UR trials, the distractors consist of one schema-consistent distractor (i.e., an NR object from the same group) and one schema-inconsistent distractor (i.e., an RR object from the same group). In NR trials, since the target is a typical, schema-consistent NR object, both distractors are schema-inconsistent (i.e., RR and UR objects from the same group). The cue and key visual components remain unchanged within each group of three objects (e.g., the same “rabbit ear” feature), ensuring that the target and distractors share identical diagnostic features and maintain consistency in primary perceptual overlap. All stimuli were presented in a standardized format (common background/canvas and comparable scaling/centering). In the object selection display, the three options were presented using the same spatial template and their positions were counterbalanced across trials to minimize systematic location-driven biases in early orienting and first-fixation latency. A “new” response does not require a second-stage reaction and proceeds directly to the next trial. Regardless of the response, the new cue advances to the next trial. Stimulus presentation and response recording were conducted using E-Prime 2.0 software (http://www.pstnet.com/eprime.cfm, accessed on 4 May 2023; RRID: SCR_009567).

### 2.4. Acquisition of Eye Movement Data

Eye movement data were collected using an EyeLink 1000 Plus eye tracker, manufactured by SR Research. A desktop recording mode was employed, which recorded only monocular fixation information. The right eye was recorded by default. If the tracking quality of the right eye was poor, then the left eye was recorded instead. The sampling rate was set to 500 Hz. Participants’ heads were stabilized with a chin rest, and they were seated in front of the monitor at a viewing distance of approximately 60 cm. The stimuli were presented on a 23-inch LCD monitor with a resolution of 1920 × 1080 pixels. The monitor was controlled by E-Prime software, which also synchronized and sent event markers to the eye-tracking system.

Before the formal experiment began, a standard nine-point fixation calibration and validation was performed for each participant. If the validation error exceeded the preset threshold or significant spatial deviation occurred, the process was recalibrated. The experiment consisted of an encoding phase and a retrieval phase. Before each phase began, a central fixation point was presented and drift correction was performed to ensure the spatial accuracy of the fixation position. Eye movement recordings included instantaneous fixation positions, pupil diameter, and trigger markers corresponding to experimental events. The system automatically marked blinks and signal losses and either removed or interpolated them during subsequent preprocessing stages.

### 2.5. Analysis of Subjective Ratings

For each participant, the ratings of surprise and unreasonableness were averaged within each condition (NR, RR, and UR). Age differences were examined using a 2 (age: young vs. older) × 3 (condition: NR, RR, and UR) mixed repeated measures analysis of variance (RM-ANOVA) in R 4.4.1 (https://cran.r-project.org/) with the “bruceR” package. When the sphericity test was violated, results were corrected using the Greenhouse–Geisser method.

### 2.6. Analysis of Memory Performance

Participants’ cue memory and object memory were assessed. Cue memory was quantified using the d′ value: d′ = Z(hit rate) − Z(false alarm rate). Object memory included two types of indicators: true memory and false memory. True object memory was calculated as the number of correctly identified objects divided by the number of object selection trials (calculated separately for each condition). False object memory was decomposed into two theory-driven categories based on the type of distractor selected: schema-consistent false memory and schema-inconsistent false memory. Schema-consistent false memory was defined as the proportion of trials in which participants selected NR objects (i.e., schema-consistent distractors) when the target was a restructured object. This metric was calculated separately for the RR and UR conditions using the following formula: Schema-consistent false memory = (number of times NR distractors were selected)/(number of object selection trials in that condition). Schema-inconsistent false memory was used to quantify cross-type confusion between restructured objects: in the RR condition, it referred to the proportion of trials in which participants selected UR objects; in the UR condition, it referred to the proportion of trials in which participants selected RR objects. For example, in the RR condition, if a participant completed 16 selection trials, with 2 selections of NR distractors and 1 selection of a UR distractor, the schema-consistent false memory would be 2/16 = 12.5%, and the schema-inconsistent false memory would be 1/16 = 6.25%. Data analysis employed RM-ANOVAs. For cue memory and true object memory, a 2 (age: young vs. older) × 3 (condition: NR, RR, and UR) RM-ANOVA was conducted. For false memory within the RR and UR conditions, a separate 2 (age: young vs. older) × 2 (false memory type: schema-consistent vs. schema-inconsistent) RM-ANOVA was performed.

In addition to memory accuracy metrics, reaction times (RTs) were analyzed to quantify processing efficiency at various levels of schema deviation. Cue-decision reaction time was defined as the delay, in seconds, from the cue’s appearance to the “old/new” judgment. Object selection reaction time was defined as the delay from display of three alternative objects to selection response in trials where participants judged the cue as “old.” The reaction times for the cue-decision phase and the object selection phase were analyzed separately using a 2 (age: young vs. older) × 3 (condition: NR, RR, UR) RM-ANOVA.

### 2.7. Analysis of Eye Movement Data

Eye-tracking preprocessing and metric extraction were performed in R using the eyelinkReader [40] to import EyeLink EDF files and extract fixation events and experiment messages. Using EyeLink Data Viewer-compatible demarcation messages (TRIALID as trial onset and TRIAL_RESULT as trial offset), trials were segmented and then merged with behavioral trial tables. Only trials from the retrieval phase were retained. Each retrieval display contained three predefined rectangular areas of interest (AOIs) that corresponded to the three object types (NR, RR, and UR). AOI boundaries were stored as pixel-coordinate rectangles (xmin, xmax, ymin, ymax) in stimulus-image space, with no additional padding or margins beyond the manually drawn rectangles. For each trial, the target AOI was defined as the AOI whose label matched the trial condition; the remaining AOIs were treated as competitive AOIs.

To reduce noise from micro-events and parsing artifacts, fixations shorter than 60 ms were excluded, consistent with common practice in fixation-based analyses [41,42]. When binocular recordings were available, a single eye per participant was analyzed using a fixed priority rule to maintain consistency within subjects. The fixation coordinates were mapped from screen space to stimulus-image space under the assumption of a centered presentation at the stimulus’ native resolution (i.e., centered without scaling). Trial-level quality control excluded trials with substantial off-stimulus gaze. For each trial, we computed the proportion of fixations and total fixation time within the stimulus area. Trials were retained only if both were ≥0.50. Table S1 shows the descriptive statistics of the number of trials included in the analysis for young and older adults separately for each condition (NR, RR, and UR), providing transparency regarding potential age- and condition-related differences in trial retention. To quantify potential retention differences, we analyzed the number of usable eye-tracking trials using an age × condition RM-ANOVA. Valid trial counts revealed significant main effects of age, *F*(1, 53) = 4.22, *p* = 0.05, *ηp*^2^ = 0.07, 90% CI = [0.00, 0.21], and condition, *F*(1.96, 104.10) = 6.20, *p* < 0.01, *ηp*^2^ = 0.11, 90% CI = [0.02, 0.20], but the age × condition interaction was not significant, *F*(1.96, 104.10) = 2.34, *p* = 0.10, *ηp*^2^ = 0.04, 90% CI = [0.00, 0.11]. Descriptively, young adults showed relatively stable trial retention across conditions (YA: NR *M* = 17.77, *SD* = 2.32; RR *M* = 17.77, *SD* = 1.90; UR *M* = 17.27, *SD* = 2.60), whereas older adults retained fewer trials overall and particularly in the restructured conditions (OA: NR *M* = 17.14, *SD* = 2.40; RR *M* = 16.00, *SD* = 3.54; UR *M* = 15.52, *SD* = 3.53). Bonferroni-adjusted follow-up tests (collapsed across age group) indicated that valid trial counts were higher in NR than UR, *t*(53) = 3.33, *p* < 0.01, *d* = 0.48, 95% CI = [0.12, 0.84], whereas NR did not differ from RR, *t*(53) = 2.01, *p* = 0.15, *d* = 0.26, 95% CI = [0.00, 0.57], and RR did not differ from UR, *t*(53) = 1.63, *p* = 0.33, *d* = 0.22, 95% CI = [0.00, 0.56]. Consistent with the main effect of age, young adults retained more usable trials than older adults averaged across conditions, *t*(53) = 2.05, *p* = 0.05, *d* = 0.63, 95% CI = [0.01, 1.24]. For the retained trials, we ordered the fixation sequences temporally and computed the eye-movement metrics at the trial × AOI level. Participant means at the condition level were computed by averaging across retained trials.

Six eye-movement metrics were analyzed in the present study. Target viewing proportion indexes the distribution of overt attention to the target AOI. Early target viewing proportion operationalizes early orienting to the target based on initial fixation evidence. First fixation latency reflects the speed of attentional orienting to the target. First fixation duration and mean fixation duration capture early and sustained processing demands, respectively. Finally, revisit rate indexes repeated sampling or returns to an AOI, which is a hallmark of competitive evaluation or re-checking (see Table S2 for details) [42,43]. These indices are widely used to study how memory guides gaze during retrieval and how such gaze–memory coupling may vary with age [44,45,46,47].

To compare age differences in eye-movement metrics, a 2 (age: young vs. older) × 3 (condition: NR, RR, UR) RM-ANOVA was conducted for the six key eye-movement metrics. Given the study’s focus on competition and selection among object types during retrieval, the primary analyses targeted the target AOI metrics (i.e., condition = AOI). After controlling for the number of valid trials as a covariate, the main results remained highly consistent (Table S3). This provides transparency that the direction and inferential conclusions of the key age × condition effects were unchanged after accounting for trial availability. Because these eye-movement metrics are partially dependent and were selected a priori as theoretically motivated indices intended to jointly characterize related components of retrieval dynamics (e.g., early orienting and sustained evaluation), we interpret the gaze results as a coherent pattern across measures rather than treating any single metric in isolation, and we report effect sizes throughout. For transparency, we additionally conducted a sensitivity analysis applying Benjamini–Hochberg false discovery rate (FDR) correction across the eye-movement metrics for each omnibus effect (i.e., the main effects and the age × condition interaction); the adjusted results were highly consistent with the uncorrected analyses, and the substantive conclusions remained unchanged (see Table S4 for FDR-adjusted *p*-values).

Because eye-movement metrics are inherently trial-varying and trial counts differed by age and condition, we additionally conducted trial-level mixed-effects models for each eye-movement outcome, treating trial as the observational unit and including participant-specific random intercepts and random slopes for condition. The trial-level results converge with the RM-ANOVA pattern of effects (see Table S5).

### 2.8. Representational Similarity Analysis

Representational similarity analysis (RSA) assesses whether two sources of data share a similar relational structure across experimental conditions. Here, RSA was used to test whether participants’ memory-choice structure across NR/RR/UR conditions corresponds to the gaze-metric structure across the same condition space during the retrieval phase (Figure 1c). Conceptually, higher eye–memory similarity indicates a tighter correspondence between how visual attention was allocated during retrieval and the pattern of subsequent object seltctions, consistent with stronger gaze–memory coupling.

We implemented the RSA in three steps. All analyses were performed in R. First, at the memory level, we constructed a behavioral representational matrix for each participant based on their three-alternative forced-choice responses during retrieval. For each participant, we aggregated the choice responses across the three conditions (NR, RR, and UR) to create a 3 × 3 matrix of choice proportions. The columns corresponded to the condition categories of the trials (NR, RR, and UR), the rows corresponded to the categories chosen by the participant during retrieval, and each cell represented the proportion of responses for choosing a specific category when the condition was that same category. The diagonal elements reflected the proportion of correct source judgments, and the off-diagonal elements reflected the proportion of misclassifying trials into other categories. This captured the inter-condition confusion structure during retrieval. Note that this 3 × 3 matrix describes participants’ choice tendencies under different true conditions. Each cell represents the relative proportion of category choices. Therefore, it is not equivalent to confusion counts based on trial numbers or classifier performance metrics. For simplicity, this paper refers to it as the “choice proportion matrix.”

Second, at the eye-movement level, we constructed subject-level representational matrices for each eye-movement metric. The analyses were included only records that provided complete condition information and met data quality requirements. These matrices had a 3 × 3 structure, with cells defined by condition (NR, RR, or UR) and area of interest (AOI: NR, RR, or UR). The cells were filled with the aggregated eye-movement metric values for the corresponding conditions and AOIs (including target and competitive AOIs) for each participant. This yielded a 3 × 3 eye-movement representational matrix for each participant and eye-movement metric.

Third, RSA similarity calculations were performed at the participant level. Both the 3 × 3 choice proportion matrix and each 3 × 3 eye-movement matrix were vectorized using the same fixed, pre-specified column-wise rule, such that entries were read top-to-bottom within each column and left-to-right across columns. This ensured an identical element ordering across the two representational spaces. For each participant and eye-movement metric, Spearman’s rank correlation (ρ) was used to compute the correlation between the “memory vector” and the “eye-movement vector,” and this correlation served as the similarity measure. To facilitate between-group inference, all ρ values were subjected to a Fisher r-to-z transformation. Between-group comparisons were conducted using Welch’s corrected independent-samples *t* tests on the Fisher-z values to examine differences in RSA similarity between young and older adults for each eye-movement metric. To address the multiple-comparisons issue arising from testing six eye-movement metrics separately, the Benjamini–Hochberg FDR method was applied to correct *p* values.

### 2.9. Robustness Analysis of RSA

To assess the robustness of the RSA findings, we conducted two complementary sensitivity analyses. First, we recomputed eye–memory similarity using Kendall’s tau, thereby reducing dependence on the specific choice of rank-correlation estimator. Group differences were evaluated using a nonparametric permutation test with 10,000 permutations, providing inference that does not rely on standard parametric assumptions.

Second, to further improve robustness to unequal numbers of usable trials, we additionally implemented a trial-level bootstrap RSA procedure. Memory-choice trials were first paired with eye-movement trials by image identity within participant and condition, and paired trials with missing eye-movement values were removed. We then determined a fixed resampling size (*N* = 12) based on the median number of quality-controlled paired trials across all participants, conditions and eye-movement metrics, and used this fixed size for resampling within each condition. For each participant and eye-movement metric, we performed 100 bootstrap resamples with replacement. In each resample, we reconstructed the participant’s 3 × 3 choice proportion matrix and the corresponding 3 × 3 eye-movement matrix from the resampled trial pairs, computed Spearman’s ρ between the two vectorized matrices, and applied Fisher r-to-z transformation. The participant’s bootstrap-based RSA estimate for a given metric was defined as the mean Fisher-z similarity across the 100 resamples. Group-level inference was then conducted on these participant-level bootstrap-mean Fisher-z values. Specifically, for each eye-movement metric, we compared young and older adults using Welch’s corrected independent-samples *t* tests. To address the multiple-comparisons issue arising from testing six eye-movement metrics separately, the Benjamini–Hochberg FDR method was applied.

### 2.10. Transparency and Reproducibility

Our approach to multiplicity is aligned with the inferential level of each test family. For post hoc pairwise comparisons within a given RM-ANOVA, we use Bonferroni adjustment to control the family-wise error rate for a small, predefined set of contrasts. For inference across the six eye-movement metrics, we additionally report Benjamini–Hochberg FDR correction as a sensitivity check, because these metrics are partially dependent and are interpreted as a converging set of theoretically motivated measures.

To improve analytic transparency and reproducibility, we provide an end-to-end record of the analysis workflow. All data analysis and visualization procedures are implemented in R scripts that reproduce the reported results. The full analysis code is available on the Open Science Framework (OSF; https://osf.io/m5hz2/files/osfstorage, accessed on 24 January 2026).

Finally, Table S8 lists the main R packages used in the analyses together with their version numbers, which supports recreation of the computational environment.

## 3. Results

We first report subjective ratings (surprise and unreasonableness) as a manipulation check. We then present behavioral memory outcomes, focusing on the age × condition interaction in true object memory and age differences in schema-consistent versus schema-inconsistent false memory. Next, we report response time measures as complementary evidence. We then describe eye-movement indices of retrieval dynamics (early target viewing and fixation duration measures, alongside additional gaze metrics), followed by RSA tests quantifying eye–memory representational correspondence across gaze metrics (FDR-corrected).

### 3.1. Age Differences in Subjective Ratings

As a manipulation check (secondary outcome), we examined whether surprise and unreasonableness ratings differentiated conditions and whether these effects varied by age.

**Figure 2:**
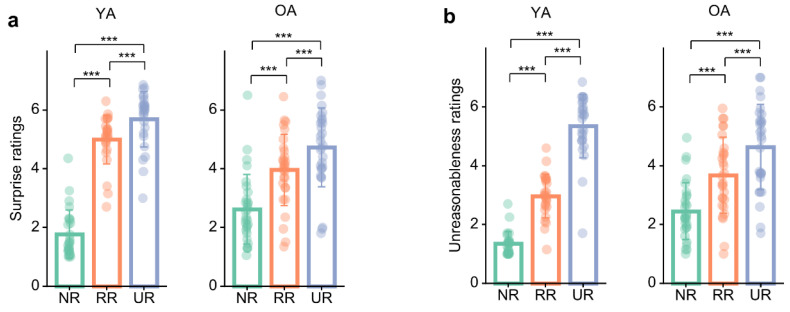
(**a**) Age differences in surprise ratings. (**b**) Age differences in unreasonableness ratings. The bars represent the mean, the dots represent the individual data values, and the error bars represent the mean ± 1 standard deviation (SD). YA, young adult; OA, older adult; NR, non-restructured; RR, reasonably restructured; UR, unreasonably restructured; *** *p* < 0.001.

Surprise ratings revealed a significant age × condition interaction, *F*(1.43, 75.91) = 34.93, *p* < 0.001, *η_p_*^2^ = 0.40, 90% CI = [0.26, 0.51] (Figure 2a), indicating that the magnitude of condition differences varied by age group. There was also a significant main effect of condition, *F*(1.43, 75.91) = 306.71, *p* < 0.001, *η_p_*^2^ = 0.85, 90% CI = [0.80, 0.89]. The main effect of age was not significant, *F*(1, 53) = 2.27, *p* = 0.138, *η_p_*^2^ = 0.04, 90% CI = [0.00, 0.16]. Descriptively, surprise increased monotonically with schema deviation in both age groups (NR < RR < UR), but ratings were more differentiated in young adults (YA: NR *M* = 1.78, *SD* = 0.82; RR *M* = 4.99, *SD* = 0.83; UR *M* = 5.68, *SD* = 0.94) than in older adults (OA: NR *M* = 2.62, *SD* = 1.18; RR *M* = 3.96, *SD* = 1.21; UR *M* = 4.73, *SD* = 1.34), consistent with the interaction reflecting more “compressed” condition differences in older adults. Bonferroni-adjusted follow-up tests within each age group confirmed this pattern. For young adults, surprise was higher in RR than NR, *t*(53) = 19.40, *p* < 0.001, *d* = 3.46, 95% CI = [3.02, 3.90], higher in UR than NR, *t*(53) = 16.70, *p* < 0.001, *d* = 4.20, 95% CI = [3.58, 4.82], and higher in UR than RR, *t*(53) = 4.93, *p* < 0.001, *d* = 0.74, 95% CI = [0.37, 1.11]. For older adults, surprise was higher in RR than NR, *t*(53) = 8.56, *p* < 0.001, *d* = 1.45, 95% CI = [1.03, 1.86], higher in UR than NR, *t*(53) = 9.54, *p* < 0.001, *d* = 2.27, 95% CI = [1.68, 2.86], and higher in UR than RR, *t*(53) = 5.81, *p* < 0.001, *d* = 0.83, 95% CI = [0.47, 1.18].

Unreasonableness ratings revealed a significant age × condition interaction, *F*(1.53, 80.83) = 26.76, *p* < 0.001, *η_p_*^2^ = 0.34, 90% CI = [0.20, 0.45] (Figure 2b), indicating that the magnitude of condition differences varied by age group. There was also a significant main effect of condition, *F*(1.53, 80.83) = 284.36, *p* < 0.001, *η_p_*^2^ = 0.84, 90% CI = [0.79, 0.88]. The main effect of age was not significant, *F*(1, 53) = 2.24, *p* = 0.141, *η_p_*^2^ = 0.04, 90% CI = [0.00, 0.16]. Descriptively, ratings increased monotonically with schema deviation in both age groups (NR < RR < UR), but this increase was more pronounced in young adults (YA: NR *M* = 1.35, *SD* = 0.41; RR *M* = 2.97, *SD* = 0.74; UR *M* = 5.35, *SD* = 1.08) than in older adults (OA: NR *M* = 2.45, *SD* = 0.97; RR *M* = 3.67, *SD* = 1.29; UR *M* = 4.64, *SD* = 1.45), consistent with the significant interaction indicating more “compressed” differentiation across conditions in older adults. Bonferroni-adjusted follow-up tests within each age group showed that, for young adults, RR ratings exceeded NR, *t*(53) = 10.25, *p* < 0.001, *d* = 1.70, 95% CI = [1.29, 2.11], UR exceeded NR, *t*(53) = 16.98, *p* < 0.001, *d* = 4.20, 95% CI = [3.58, 4.81], and UR exceeded RR, *t*(53) = 14.63, *p* < 0.001, *d* = 2.50, 95% CI = [2.08, 2.92]. The same ordinal pattern was observed in older adults: RR > NR, *t*(53) = 8.22, *p* < 0.001, *d* = 1.29, 95% CI = [0.90, 1.68]; UR > NR, *t*(53) = 9.83, *p* < 0.001, *d* = 2.30, 95% CI = [1.72, 2.88]; and UR > RR, *t*(53) = 6.26, *p* < 0.001, *d* = 1.01, 95% CI = [0.61, 1.41].

### 3.2. Age Differences in True Memory Performance

As primary behavioral outcomes, we assessed cue memory and true object memory across conditions, with particular focus on the age × condition interaction in true object memory.

**Figure 3:**
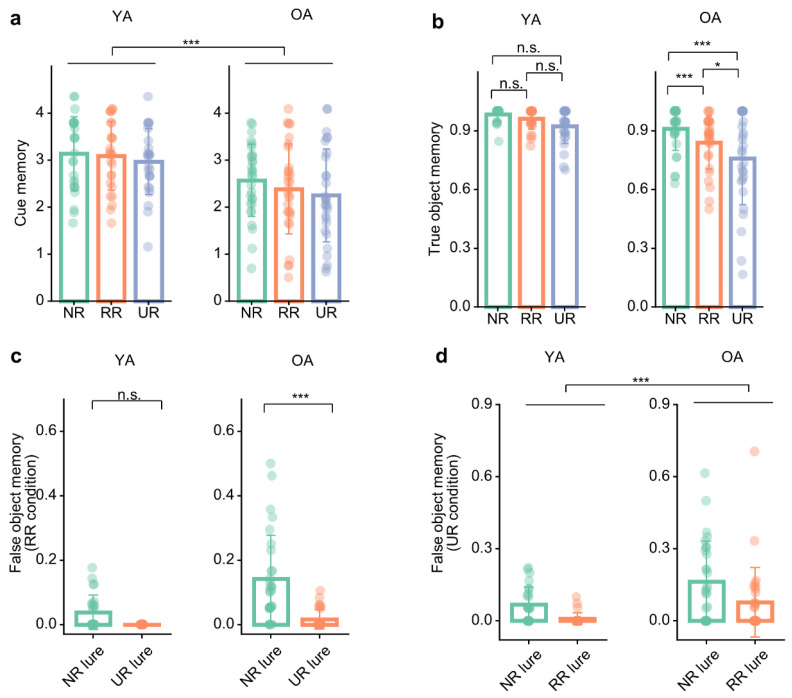
(**a**) Age differences in cue memory. (**b**) Age differences in true object memory. (**c**) Age differences in false object memory in the RR condition. (**d**) Age differences in false object memory in the UR condition. The bars represent the mean, the dots represent the individual data values, and the error bars represent the mean ± 1 standard deviation (SD). YA, young adult; OA, older adult; NR, non-restructured; RR, reasonably restructured; UR, unreasonably restructured; * *p* < 0.05; *** *p* < 0.001; n.s., not significant.

Cue memory showed a significant main effect of age, *F*(1, 53) = 9.69, *p* = 0.003, *η_p_*^2^ = 0.16, 90% CI = [0.04, 0.31] (Figure 3a), with young adults showing overall better cue memory than older adults. Collapsing across conditions, young adults outperformed older adults, *t*(53) = 3.11, *p* = 0.003, *d* = 1.42, 95% CI = [0.51, 2.34]. There was also a significant main effect of condition, *F*(1.91, 101.37) = 7.21, *p* = 0.001, *η_p_*^2^ = 0.12, 90% CI = [0.03, 0.22]. Bonferroni-adjusted pairwise comparisons across conditions showed that cue memory in the NR condition was significantly higher than in the UR condition, *t*(53) = 3.47, *p* = 0.003, *d* = 0.52, 95% CI = [0.15, 0.89], whereas the differences between NR and RR, *t*(53) = 1.95, *p* = 0.168, *d* = 0.24, 95% CI = [−0.06, 0.55], and between RR and UR, *t*(53) = 2.04, *p* = 0.138, *d* = 0.28, 95% CI = [−0.06, 0.61], were not statistically significant. The age × condition interaction was not significant, *F*(1.91, 101.37) = 0.81, *p* = 0.442, *η_p_*^2^ = 0.02, 90% CI = [0.00, 0.06], indicating that the pattern of condition effects on cue memory was similar for young and older adults.

True object memory revealed a significant age × condition interaction, *F*(1.73, 91.43) = 3.33, *p* = 0.047, *η_p_*^2^ = 0.06, 90% CI = [0.00, 0.15] (Figure 3b). Post hoc comparisons showed that, in young adults, true object memory did not differ reliably between the NR (*M* = 0.99, *SD* = 0.04) and RR (*M* = 0.96, *SD* = 0.05) conditions, *t*(53) = 1.18, *p* = 0.736, *d* = 0.18, 95% CI = [−0.20, 0.57], between the NR and UR (UR: *M* = 0.92, *SD* = 0.09) conditions, *t*(53) = 2.18, *p* = 0.101, *d* = 0.49, 95% CI = [−0.07, 1.04], or between the RR and UR conditions, *t*(53) = 1.47, *p* = 0.443, *d* = 0.31, 95% CI = [−0.21, 0.82]. In contrast, older adults’ true object memory in the NR (*M* = 0.91, *SD* = 0.11) condition was significantly higher than that in the RR (*M* = 0.84, *SD* = 0.14) and UR (*M* = 0.76, *SD* = 0.24) conditions (NR vs. RR: *t*(53) = 3.72, *p* = 0.001, *d* = 0.55, 95% CI = [0.18, 0.91]; NR vs. UR: *t*(53) = 5.62, *p* < 0.001, *d* = 1.19, 95% CI = [0.67, 1.72]). In older adults, true object memory in the RR condition was also significantly higher than that in the UR condition, *t*(53) = 3.28, *p* = 0.006, *d* = 0.65, 95% CI = [0.16, 1.14]. The main effects of age, *F*(1, 53) = 16.74, *p* < 0.001, *η_p_*^2^ = 0.24, 90% CI = [0.09, 0.39], and condition, *F*(1.73, 91.43) = 19.11, *p* < 0.001, *η_p_*^2^ = 0.27, 90% CI = [0.14, 0.38], were also significant.

### 3.3. Age Differences in False Memory Performance

As a primary test of schema-related memory errors, we examined schema-consistent versus schema-inconsistent false memory in the RR and UR conditions.

In the RR condition, the interaction of age × false-memory type was significant, *F*(1, 53) = 9.03, *p* = 0.004, *η_p_*^2^ = 0.15, 90% CI = [0.03, 0.30] (Figure 3c). Post hoc comparisons showed that older adults exhibited significantly higher schema-consistent false memory (*M* = 0.14, *SD* = 0.14) than schema-inconsistent false memory (*M* = 0.02, *SD* = 0.03), *t*(53) = 6.26, *p* < 0.001, *d* = 1.17, 95% CI = [0.80, 1.55], whereas young adults showed no significant difference between schema-consistent (*M* = 0.04, *SD* = 0.05) and schema-inconsistent false memory (*M* = 0.00, *SD* = 0.00), *t*(53) = 1.79, *p* = 0.080, *d* = 0.35, 95% CI = [−0.04, 0.75]. Additionally, the main effects of age, *F*(1, 53) = 18.03, *p* < 0.001, *η_p_*^2^ = 0.25, 90% CI = [0.10, 0.41], and false-memory type, *F*(1, 53) = 31.33, *p* < 0.001, *η_p_*^2^ = 0.37, 90% CI = [0.21, 0.51], were also significant.

In the UR condition, the main effect of age was significant, *F*(1, 53) = 11.04, *p* = 0.002, *η_p_*^2^ = 0.17, 90% CI = [0.05, 0.32] (Figure 3d), indicating higher overall false memory in older adults than young adults, *t*(53) = 3.32, *p* = 0.002, *d* = 0.53, 95% CI = [0.21, 0.84]. The main effect of false-memory type was also significant, *F*(1, 53) = 11.65, *p* = 0.001, *η_p_*^2^ = 0.18, 90% CI = [0.05, 0.33], with schema-consistent false memory (YA: *M* = 0.07, *SD* = 0.07; OA: *M* = 0.16, *SD* = 0.17) being higher than schema-inconsistent false memory (YA: *M* = 0.01, *SD* = 0.03; OA: *M* = 0.08, *SD* = 0.15), *t*(53) = 3.41, *p* = 0.001, *d* = 0.47, 95% CI = [0.19, 0.74]. However, the interaction of age × false-memory type was not significant, *F*(1, 53) = 0.40, *p* = 0.531, *η_p_*^2^ = 0.01, 90% CI = [0.00, 0.09].

### 3.4. Age Differences in Response Time

As a secondary analysis, we analyzed cue-decision and object-selection response times to characterize processing-speed and decision-time differences across age groups.

**Figure 4:**
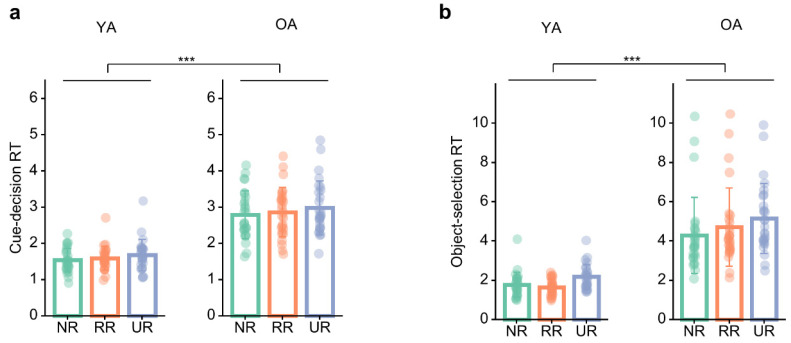
(**a**) Age differences in cue-decision response time. (**b**) Age differences in object-selection response time. The bars represent the mean, the dots represent the individual data values, and the error bars represent the mean ± 1 standard deviation (SD). YA, young adult; OA, older adult; NR, non-restructured; RR, reasonably restructured; UR, unreasonably restructured; *** *p* < 0.001.

Cue-decision RTs showed a significant main effect of age, *F*(1, 53) = 82.86, *p* < 0.001, *η_p_*^2^ = 0.61, 90% CI = [0.47, 0.71] (Figure 4a), with older adults responding more slowly overall than young adults. Collapsing across conditions, older adults exhibited longer cue RTs than young adults, *t*(53) = 9.10, *p* < 0.001, *d* = 3.49, 95% CI = [2.72, 4.26]. There was also a significant main effect of condition, *F*(1.89, 100.26) = 7.14, *p* = 0.002, *η_p_*^2^ = 0.12, 90% CI = [0.03, 0.22]. Bonferroni-adjusted pairwise comparisons across conditions showed that cue-decision RTs were significantly longer in the UR condition than in the NR condition, *t*(53) = 3.90, *p* < 0.001, *d* = 0.51, 95% CI = [0.19, 0.82], whereas the differences between RR and NR (*t*(53) = 1.28, *p* = 0.616, *d* = 0.16, 95% CI = [−0.15, 0.48]) and between UR and RR (*t*(53) = 2.26, *p* = 0.085, *d* = 0.34, 95% CI = [−0.03, 0.72]) were not statistically significant. The age × condition interaction was not significant, *F*(1.89, 100.26) = 0.25, *p* = 0.770, *η_p_*^2^ = 0.01, 90% CI = [0.00, 0.03], indicating that the pattern of condition-related cue-decision RT differences was similar for young and older adults.

For object-selection RT, there was a significant main effect of age, *F*(1, 53) = 68.53, *p* < 0.001, *η_p_*^2^ = 0.56, 90% CI = [0.42, 0.67] (Figure 4b), with older adults showing longer RTs than young adults. Collapsing across conditions, older adults responded more slowly than young adults, *t*(53) = 8.28, *p* < 0.001, *d* = 2.47, 95% CI = [1.87, 3.07]. The main effect of condition was also significant, *F*(1.61, 85.36) = 11.09, *p* < 0.001, *η_p_*^2^ = 0.17, 90% CI = [0.06, 0.29]. Bonferroni-adjusted post hoc tests showed that object-selection RTs were longer in the UR condition than in both the NR condition, *t*(53) = 4.30, *p* < 0.001, *d* = 0.62, 95% CI = [0.26, 0.98], and the RR condition, *t*(53) = 4.60, *p* < 0.001, *d* = 0.46, 95% CI = [0.21, 0.70], whereas the RR vs. NR comparison was not significant, *t*(53) = 1.03, *p* = 0.921, *d* = 0.16, 95% CI = [−0.23, 0.98]. The age × condition interaction was not significant, *F*(1.61, 85.36) = 2.30, *p* = 0.117, *η_p_*^2^ = 0.04, 90% CI = [0.00, 0.12], suggesting that the condition effect on object-selection RT did not differ reliably between age groups.

### 3.5. Age Differences in Eye Movement

To test our primary gaze hypotheses, we analyzed early orienting (early target viewing) and evaluation indices (first/mean fixation duration) across conditions. Additional gaze measures (target viewing proportion, first fixation latency, and revisit rate) are reported as complementary.

**Figure 5:**
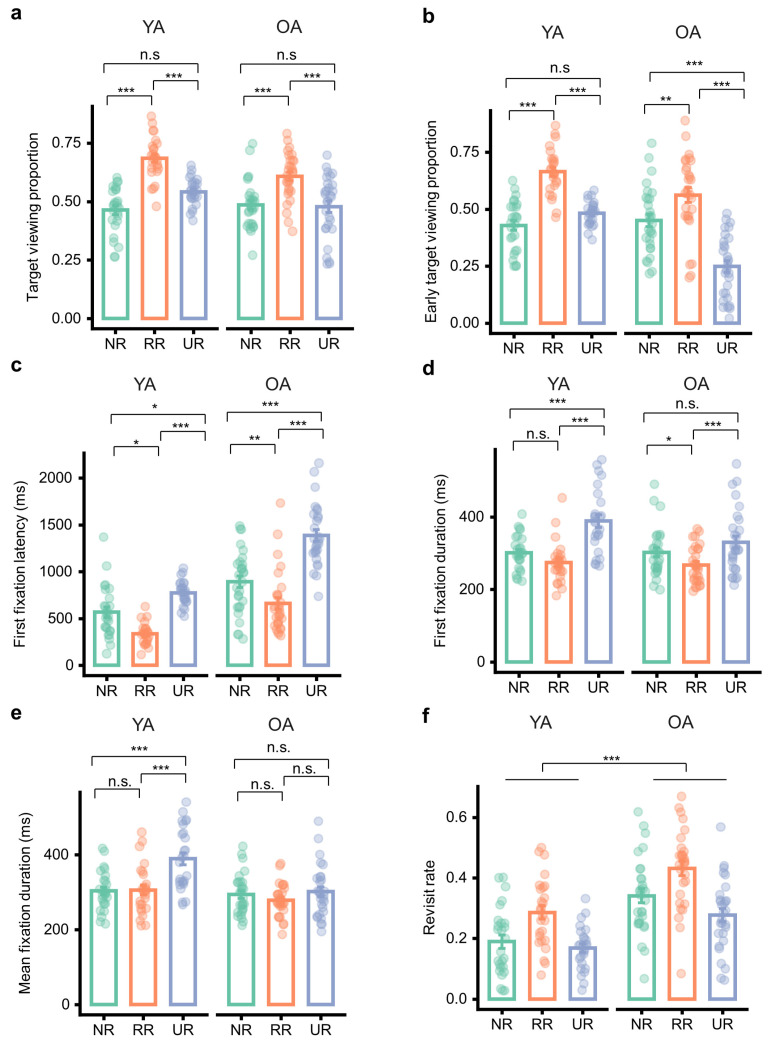
(**a**) Age differences in target viewing proportion. (**b**) Age differences in early target viewing proportion. (**c**) Age differences in first fixation latency. (**d**) Age differences in first fixation duration. (**e**) Age differences in mean fixation duration. (**f**) Age differences in revisit rate. The bars represent the mean, the dots represent the individual data values, and the error bars represent the mean ± 1 standard deviation (SD). YA, young adult; OA, older adult; NR, non-restructured; RR, reasonably restructured; UR, unreasonably restructured; * *p* < 0.05; ** *p* < 0.01; *** *p* < 0.001; n.s., not significant.

Target viewing proportion showed a significant age × condition interaction, *F*(1.70, 90.26) = 3.80, *p* = 0.032, *η_p_*^2^ = 0.07, 90% CI = [0.003, 0.16] (Figure 5a), alongside significant main effects of age, *F*(1, 53) = 7.54, *p* = 0.008, *η_p_*^2^ = 0.13, 90% CI = [0.02, 0.27], and condition, *F*(1.70, 90.26) = 42.19, *p* < 0.001, *η_p_*^2^ = 0.44, 90% CI = [0.32, 0.54]. Descriptively, target viewing proportion peaked in the RR condition for both age groups, but the condition modulation was more pronounced in young adults (YA: NR *M* = 0.46, *SD* = 0.10; RR *M* = 0.69, *SD* = 0.09; UR *M* = 0.54, *SD* = 0.06) than in older adults (OA: NR *M* = 0.49, *SD* = 0.10; RR *M* = 0.61, *SD* = 0.10; UR *M* = 0.48, *SD* = 0.13), consistent with the interaction indicating age-related differences in the magnitude/pattern of condition effects. Bonferroni-adjusted follow-up tests within each age group clarified this pattern. For young adults, target viewing proportion was higher in RR than NR, *t*(53) = 7.94, *p* < 0.001, *d* = 1.53, 95% CI = [1.06, 2.00], and higher in RR than UR, *t*(53) = 6.11, *p* < 0.001, *d* = 0.99, 95% CI = [0.59, 1.39], whereas the UR vs. NR difference was not significant, *t*(53) = 2.33, *p* = 0.070, *d* = 0.54, 95% CI = [−0.03, 1.12]. For older adults, target viewing proportion was higher in RR than NR, *t*(53) = 4.60, *p* < 0.001, *d* = 0.84, 95% CI = [0.39, 1.29], and higher in RR than UR, *t*(53) = 5.86, *p* < 0.001, *d* = 0.90, 95% CI = [0.52, 1.27], whereas the NR vs. UR difference was not significant, *t*(53) = 0.26, *p* = 1.000, *d* = 0.06, 95% CI = [−0.49, 0.60].

Early target viewing proportion exhibited a significant age × condition interaction, *F*(1.71, 90.77) = 11.70, *p* < 0.001, *η_p_*^2^ = 0.18, 90% CI = [0.07, 0.29] (Figure 5b), together with strong main effects of age, *F*(1, 53) = 40.60, *p* < 0.001, *η_p_*^2^ = 0.43, 90% CI = [0.27, 0.56], and condition, *F*(1.71, 90.77) = 46.97, *p* < 0.001, *η_p_*^2^ = 0.47, 90% CI = [0.35, 0.57]. Descriptively, young adults showed elevated early target viewing in the RR condition relative to NR and UR (YA: NR *M* = 0.43, *SD* = 0.11; RR *M* = 0.66, *SD* = 0.10; UR *M* = 0.48, *SD* = 0.05), whereas older adults showed a marked reduction in early target viewing in the UR condition (OA: NR *M* = 0.45, *SD* = 0.15; RR *M* = 0.56, *SD* = 0.17; UR *M* = 0.25, *SD* = 0.14), consistent with the interaction indicating divergent condition modulation across age groups. Bonferroni-adjusted simple comparisons within each age group further characterized this pattern. For young adults, early target viewing proportion was higher in RR than NR, *t*(53) = 5.31, *p* < 0.001, *d* = 1.22, 95% CI = [0.65, 1.79], and higher in RR than UR, *t*(53) = 5.97, *p* < 0.001, *d* = 0.95, 95% CI = [0.55, 1.34], whereas UR did not differ significantly from NR, *t*(53) = 1.39, *p* = 0.510, *d* = 0.28, 95% CI = [−0.22, 0.77]. For older adults, early target viewing proportion was higher in RR than NR, *t*(53) = 2.69, *p* = 0.029, *d* = 0.59, 95% CI = [0.05, 1.12], higher in NR than UR, *t*(53) = 5.54, *p* < 0.001, *d* = 1.04, 95% CI = [0.58, 1.51], and higher in RR than UR, *t*(53) = 10.84, *p* < 0.001, *d* = 1.63, 95% CI = [1.26, 2.00].

First fixation latency showed a significant age × condition interaction, *F*(1.61, 85.55) = 4.92, *p* = 0.015, *η_p_*^2^ = 0.09, 90% CI = [0.01, 0.19] (Figure 5c), accompanied by significant main effects of age, *F*(1, 53) = 104.51, *p* < 0.001, *η_p_*^2^ = 0.66, 90% CI = [0.54, 0.75], and condition, *F*(1.61, 85.55) = 61.25, *p* < 0.001, *η_p_*^2^ = 0.54, 90% CI = [0.42, 0.63]. Descriptively, older adults exhibited longer first fixation latencies overall (OA: NR *M* = 892.34, *SD* = 335.44; RR *M* = 660.03, *SD* = 333.47; UR *M* = 1387.05, *SD* = 325.33) than young adults (YA: NR *M* = 567.08, *SD* = 276.11; RR *M* = 335.99, *SD* = 112.40; UR *M* = 774.37, *SD* = 128.00), with both groups showing the shortest latencies in RR and the longest in UR. Bonferroni-adjusted follow-up tests within each age group indicated that, for young adults, first fixation latency was significantly shorter in RR than NR, *t*(53) = 2.76, *p* = 0.024, *d* = 0.59, 95% CI = [0.06, 1.13], and significantly longer in UR than RR, *t*(53) = 7.93, *p* < 0.001, *d* = 1.13, 95% CI = [0.78, 1.48], whereas the UR vs. NR contrast did not reach significance, *t*(53) = 2.36, *p* = 0.066, *d* = 0.53, 95% CI = [−0.03, 1.09]. For older adults, first fixation latency was significantly shorter in RR than NR, *t*(53) = 2.93, *p* = 0.015, *d* = 0.60, 95% CI = [0.09, 1.10], and significantly longer in UR than both NR, *t*(53) = 5.96, *p* < 0.001, *d* = 1.27, 95% CI = [0.74, 1.80], and RR, *t*(53) = 13.89, *p* < 0.001, *d* = 1.87, 95% CI = [0.54, 2.20].

First fixation duration indicated a significant age × condition interaction, *F*(1.95, 103.49) = 4.73, *p* = 0.011, *η_p_*^2^ = 0.08, 90% CI = [0.01, 0.17] (Figure 5d), indicating that condition-related modulation of first fixation duration differed by age group. The main effect of condition was significant, *F*(1.95, 103.49) = 36.26, *p* < 0.001, *η_p_*^2^ = 0.41, 90% CI = [0.29, 0.50], whereas the main effect of age was not significant, *F*(1, 53) = 2.26, *p* = 0.139, *η_p_*^2^ = 0.04, 90% CI = [0.00, 0.16]. Descriptively, first fixation duration increased markedly in the UR condition for young adults (YA: NR *M* = 300.65, *SD* = 48.98; RR *M* = 273.90, *SD* = 56.30; UR *M* = 388.44, *SD* = 87.32), whereas older adults showed a comparatively attenuated increase (OA: NR *M* = 302.36, *SD* = 67.08; RR *M* = 267.53, *SD* = 51.46; UR *M* = 330.26, *SD* = 88.22), consistent with the observed interaction. Bonferroni-adjusted follow-up tests within each age group further clarified the pattern. For young adults, first fixation duration was significantly longer in UR than NR, *t*(53) = 5.36, *p* < 0.001, *d* = 1.13, 95% CI = [0.61, 1.65], and significantly longer in UR than RR, *t*(53) = 7.48, *p* < 0.001, *d* = 1.48, 95% CI = [0.99, 1.97], whereas the RR vs. NR contrast was not significant, *t*(53) = 1.87, *p* = 0.200, *d* = 0.35, 95% CI = [−0.11, 0.80]. For older adults, first fixation duration was significantly shorter in RR than NR, *t*(53) = 2.57, *p* = 0.039, *d* = 0.45, 95% CI = [0.02, 0.88], and significantly longer in UR than RR, *t*(53) = 4.33, *p* < 0.001, *d* = 0.81, 95% CI = [0.35, 1.27], whereas the UR vs. NR contrast was not statistically significant, *t*(53) = 1.80, *p* = 0.232, *d* = 0.36, 95% CI = [−0.13, 0.85].

Mean fixation duration demonstrated a significant age × condition interaction, *F*(1.94, 102.89) = 13.45, *p* < 0.001, *η_p_*^2^ = 0.20, 90% CI = [0.09, 0.31] (Figure 5e), together with significant main effects of age, *F*(1, 53) = 8.72, *p* = 0.005, *η_p_*^2^ = 0.14, 90% CI = [0.03, 0.29], and condition, *F*(1.94, 102.89) = 26.75, *p* < 0.001, *η_p_*^2^ = 0.34, 90% CI = [0.21, 0.44]. Descriptively, mean fixation duration increased substantially in the UR condition for young adults (YA: NR *M* = 303.12, *SD* = 51.99; RR *M* = 305.59, *SD* = 65.98; UR *M* = 389.06, *SD* = 82.24), whereas older adults showed comparatively modest changes across conditions (OA: NR *M* = 293.47, *SD* = 54.39; RR *M* = 278.53, *SD* = 43.00; UR *M* = 301.12, *SD* = 69.25), consistent with the interaction indicating differential condition sensitivity by age group. Bonferroni-adjusted follow-up tests within each age group further specified this pattern. For young adults, mean fixation duration was significantly longer in UR than NR, *t*(53) = 7.58, *p* < 0.001, *d* = 1.48, 95% CI = [1.00, 1.96], and significantly longer in UR than RR, *t*(53) = 6.72, *p* < 0.001, *d* = 1.44, 95% CI = [0.91, 1.96], whereas RR did not differ from NR, *t*(53) = 0.23, *p* = 1.000, *d* = 0.04, 95% CI = [−0.41, 0.50]. In older adults, none of the pairwise contrasts survived Bonferroni correction: RR did not differ from NR, *t*(53) = 1.47, *p* = 0.441, *d* = 0.26, 95% CI = [−0.17, 0.69], UR did not differ from NR, *t*(53) = 0.71, *p* = 1.000, *d* = 0.13, 95% CI = [−0.33, 0.59], and UR did not differ from RR, *t*(53) = 1.92, *p* = 0.180, *d* = 0.39, 95% CI = [−0.11,0.89].

Revisit rate showed a significant main effect of age, *F*(1, 53) = 32.86, *p* < 0.001, *η_p_*^2^ = 0.38, 90% CI = [0.22, 0.52] (Figure 5f), with older adults exhibiting a higher overall revisit rate than young adults. Collapsing across conditions, revisit rate was significantly higher in older than young adults, *t*(53) = 5.73, *p* < 0.001, *d* = 1.08, 95% CI = [0.70, 1.46]. There was also a significant main effect of condition, *F*(1.95, 103.20) = 33.11, *p* < 0.001, *η_p_*^2^ = 0.39, 90% CI = [0.26, 0.48]. Bonferroni-adjusted pairwise comparisons across conditions (collapsed across age) indicated that revisit rate was higher in RR than NR, *t*(53) = 5.08, *p* < 0.001, *d* = 0.75, 95% CI = [0.38, 1.11], higher in NR than UR, *t*(53) = 2.55, *p* = 0.041, *d* = 0.34, 95% CI = [0.01, 0.66], and higher in RR than UR, *t*(53) = 8.40, *p* < 0.001, *d* = 1.08, 95% CI = [0.77, 1.40]. The age × condition interaction was not significant, *F*(1.95, 103.20) = 0.90, *p* = 0.407, *η_p_*^2^ = 0.02, 90% CI = [0.00, 0.07], indicating that the condition effect on revisit rate was comparable across age groups.

### 3.6. Age Differences in Representational Similarity

As a primary test linking online visual sampling to retrieval decisions, we used representational similarity analysis (RSA) to quantify eye–memory correspondence across the six eye-movement metrics and compared these indices between age groups (Welch’s *t* tests; FDR-corrected).

**Figure 6:**
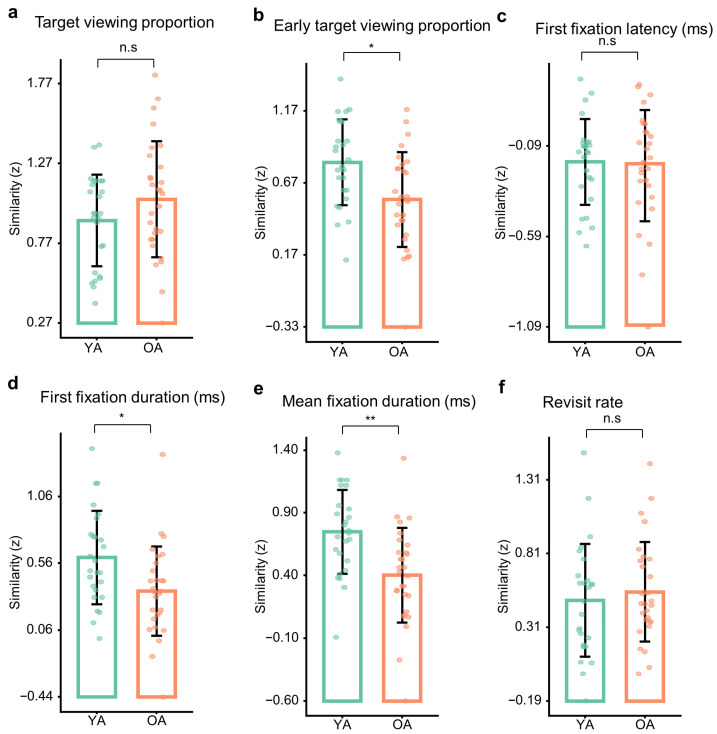
(**a**) Age differences in representational similarity between memory and target viewing proportion. (**b**) Age differences in representational similarity between memory and early target viewing proportion. (**c**) Age differences in representational similarity between memory and first fixation latency. (**d**) Age differences in representational similarity between memory and first fixation duration. (**e**) Age differences in representational similarity between memory and mean fixation duration. (**f**) Age differences in representational similarity between memory and revisit rate. The bars represent the mean, the dots represent the individual data values, and the error bars represent the mean ± 1 standard deviation (SD). YA, young adult; OA, older adult; * *p* < 0.05; ** *p* < 0.01; n.s., not significant.

After FDR correction, higher eye–memory representational correspondence in young adults was observed for first fixation duration (YA: *M* = 0.61, *SD* = 0.35; OA: *M* = 0.36, *SD* = 0.33), Welch’s *t*(51.8) = 2.72, *p* = 0.009, *p*_FDR_ = 0.018, *d* = 0.74, 95% CI = [0.21, 1.35]; mean fixation duration (YA: *M* = 0.75, *SD* = 0.34; OA: *M* = 0.40, *SD* = 0.38), Welch’s *t*(53.0) = 3.61, *p* < 0.001, *p*_FDR_ = 0.004, *d* = 0.97, 95% CI = [0.44, 1.63]; and early target viewing proportion (YA: *M* = 0.81, *SD* = 0.30; OA: *M* = 0.55, *SD* = 0.33), Welch’s *t*(53.0) = 3.05, *p* = 0.004, *p*_FDR_ = 0.011, *d* = 0.82, 95% CI = [0.31, 1.43] (Figure 6). Taken together, these effects indicate that in young adults, early attentional engagement with the target and subsequent sustained processing (indexed by fixation duration measures) more closely track the structure of later retrieval choices, consistent with tighter coupling between gaze-based evidence accumulation and memory decisions. Notably, the age group differences that survived FDR correction were medium-to-large in magnitude (*d* = 0.74–0.97), supporting the practical significance of reduced eye–memory correspondence in older adults for these theoretically motivated metrics.

No statistically significant age group differences survived FDR correction for the remaining metrics. First fixation latency did not differ between age groups (YA: *M* = −0.18, *SD* = 0.24; OA: *M* = −0.20, *SD* = 0.31), Welch’s *t*(52.0) = 0.28, *p* = 0.779, *p*_FDR_ = 0.779, *d* = 0.08, 95% CI = [−0.49, 0.60]. Target viewing proportion showed no significant age effect (YA: *M* = 0.91, *SD* = 0.29; OA: *M* = 1.05, *SD* = 0.36), Welch’s *t*(52.2) = 1.51, *p* = 0.137, *p*_FDR_ = 0.205, *d* = 0.41, 95% CI = [−0.12, 0.96]. Revisit rate also did not differ (YA: *M* = 0.50, *SD* = 0.38; OA: *M* = 0.56, *SD* = 0.34), Welch’s *t*(50.2) = 0.59, *p* = 0.559, *p*_FDR_ = 0.671, *d* = 0.16, 95% CI = [−0.36, 0.72] (Figure 6).

Robustness analyses were consistent with the primary RSA pattern (Tables S6 and S7). Using Kendall’s tau similarity with a nonparametric permutation test, significant age group differences were again observed for early target viewing proportion, first fixation duration, and mean fixation duration after FDR correction (Table S6), whereas target viewing proportion, first fixation latency, and revisit rate remained non-significant. In addition, the trial-level bootstrap RSA yielded highly consistent conclusions (Table S7). Specifically, early target viewing proportion and mean fixation duration remained significant after FDR correction in the bootstrap-based group comparisons (early target viewing: *t*(51.72) = 4.04, *p* < 0.001, *p*_FDR_ = 0.001, *d* = 1.08, 95% CI = [0.57, 1.71]; mean fixation duration: *t*(50.73) = 3.73, *p* < 0.001, *p*_FDR_ = 0.001, *d* = 1.01, 95% CI = [0.47, 1.64]). First fixation duration showed the same directional difference but did not survive FDR correction in the bootstrap analysis (*t*(41.19) = 2.18, *p* = 0.035, *p*_FDR_ = 0.069, *d* = 0.60, 95% CI = [0.13, 1.09]). Together, these sensitivity checks indicate that the primary age group differences in eye–memory correspondence are robust to the similarity estimator and inference framework, and are not attributable to unequal numbers of usable trials.

## 4. Discussion

The present study suggests that aging is associated with difficulty remembering objects that differ from a familiar schema, even when diagnostic cue information remains the same across alternatives. Behaviorally, older participants exhibited poorer cue discrimination and true object memory, with the greatest decline seen for restructured targets. Their errors were systematic. When the target was a restructured object, older adults disproportionately selected the schema-consistent NR objects, suggesting a bias toward schema-consistent lures under heightened uncertainty. Eye tracking during retrieval provided temporally resolved indices of online visual sampling under competition and further suggested that age-related differences emerged in how attention was allocated under competition. In both age groups, reasonably restructured targets elicited the most efficient orienting and the highest target viewing proportion, consistent with relatively rapid and sustained sampling of the target when schema deviation remained conceptually integrable. In contrast, unreasonably restructured targets produced the slowest orienting in both groups. Evidence for prolonged evaluative processing, however, was most apparent in young adults, who showed increased fixation duration-based indices when targets were maximally schema-deviant, whereas older adults exhibited a comparatively attenuated modulation of fixation duration across object types. Finally, representational similarity analyses showed that the correspondence between eye-movement patterns and the structure of subsequent memory choices was reduced in older adults specifically for early target viewing and fixation duration-based measures, suggesting that visual evidence sampling was less tightly coupled to the decision policy governing their object selections.

These findings align with contemporary accounts framing prior knowledge as a resource that may introduce systematic bias in cognitive aging. In our task, older adults showed poorer discrimination and a stronger tendency to choose schema-consistent objects when the target object was altered, which is consistent with an increased reliance on semantic structure and default expectations to resolve competition during the retrieval process. While this reliance may be adaptive when schematic information is diagnostic, in the present paradigm, it was accompanied by greater confusion because accurate performance required selecting item-specific details that conflicted with a well-established schema [12,33,34]. Subjective ratings provide converging evidence: both age groups preserved the intended ordering of schema deviation across conditions, but older adults showed a compressed separation among them, suggesting reduced subjective differentiation despite intact ordinal structure. This pattern is consistent with the possibility that strong priors attenuate the perceived salience of deviations. At the same time, compressed differentiation could also reflect perceptual or salience-weighting changes that reduce the diagnostic impact of deviations at retrieval. Related work on schematic congruency and prediction-based processing further suggests that aging is associated with greater weighting of top-down expectations, which can diminish the impact of schema deviation on memory while enhancing congruency-biased responding at retrieval [33,48,49,50].

The false memories produced by this task are particularly informative because they capture schema-consistent errors as well as confusion among nonmatching objects. Older participants exhibited a stronger pattern of schema-consistent false memory, disproportionately endorsing the non-restructured option when the target was a schema-deviant object. This suggests that schema-congruent lures may be more likely to serve as the default option during retrieval. This bias aligns with findings suggesting that older adults rely more on semantic structure and may have more difficulty suppressing prepotent responses when multiple candidates compete. Together, these factors may contribute to schema-consistent endorsements when item-specific evidence is inconclusive [51,52]. Crucially, the task also distinguishes between less and more integrable forms of schema deviation. Relatively better performance and more efficient gaze allocation in the reasonably restructured condition suggest that integrable schema-deviant objects can contribute diagnostic evidence for retrieval. However, non-integrable schema-deviant objects more strongly challenge evidence acquisition and evaluation, particularly in older adults [33,34].

Eye-movement measures sharpened these behavioral inferences by localizing when, during retrieval, age differences emerged. In young adults, fixation durations increased when the target was a strongly schema-deviant object, consistent with more sustained evaluation when cue-driven expectations (i.e., typical schemas) conflicted with the viewed object. Longer fixations are widely interpreted as reflecting increased processing demands and ongoing information uptake, and gaze-based sequential sampling accounts further suggest that extended dwelling can amplify evidence accumulation and bias subsequent choices [53,54,55,56,57]. In older adults, strongly schema-deviant objects instead incurred a cost in early orienting and did not elicit a comparable increase in fixation duration, suggesting reduced flexibility in adjusting online visual sampling when conflict is high and that this constraint may be evident early in the retrieval episode. From a cognitive-control perspective, such conflict-contingent adjustment is expected to support interference resolution under competition; accordingly, this pattern is compatible with accounts emphasizing age-related limitations in reactive control and interference resolution recruited after conflict detection [58,59]. Representational similarity analyses provided converging process–outcome evidence. Age differences were not uniform across gaze measures but were most pronounced during early target viewing and in correspondence with memory-choice structure based on fixation duration. Importantly, robustness analyses supported this selectivity. Using Kendall’s tau with permutation-based inference, the proportion of early target viewing and the correspondence based on fixation duration remained reliably reduced in older adults. However, the proportion of target viewing, first fixation latency, and revisit rate did not show consistent age differences. Complementary trial-level bootstrap RSA yielded consistent conclusions for early target viewing proportion and mean fixation duration after FDR correction, while the first fixation duration effect was directionally consistent but less stable under bootstrap-based multiple comparison control. This selectivity is consistent with a specific weakening of the functional linkage between early sampling, sustained evaluation, and later memory selection, rather than a general breakdown in the coupling between eye movements and memory [30,46,60].

To clarify which aspects of the data most directly support control-based versus representational fidelity accounts, we distinguish between outcome-level and process-level evidence. The behavioral profile, which includes poorer cue discrimination and true object memory (especially for restructured targets), as well as a disproportionate tendency to endorse schema-consistent lures when targets are schema-deviant, most directly supports reduced item-specific mnemonic specificity/precision (i.e., lower representational fidelity) when competing with schema-congruent alternatives [61,62]. This is consistent with the broader notion that aging is associated with increased gist/schematic responding and reduced monitoring of item-specific details [52,63]. Complementary work suggests that schema-driven biases in older adults’ memory decisions are amplified when recollection or precision is compromised [34]. In contrast, eye-movement dynamics and RSA provide temporal constraints on how evidence is sampled and utilized during retrieval. Eye movements are widely used as process measures that index moment-to-moment information sampling and can be modeled as part of the decision-formation process [64,65]. Gaze-dependent evidence-accumulation frameworks formalize the relationship between fluctuations in visual attention and subsequent choices and response dynamics [66,67]. Accordingly, stronger deviation-related modulation of fixation duration-based indices and stronger gaze-choice correspondence in young adults, alongside attenuated modulation and reduced correspondence in older adults, are compatible with age-related differences in conflict-sensitive control and adaptive adjustment under interference [68,69,70], while remaining open to non-control contributors (e.g., perceptual/oculomotor or salience-weighting factors). These accounts are not mutually exclusive. Reduced representational fidelity can increase ambiguity and competition, which may reduce the effectiveness of conflict-contingent adjustments and promote schema-biased responding. Overall, these process signatures provide mechanistic constraints, but they are not uniquely diagnostic of a specific underlying mechanism [71].

Building on this evidence mapping, these results align with models that emphasize the combined significance of representational fidelity and control demands in cognitive aging. According to this perspective, older adults may develop more cluttered or less selectively constrained event representations. This may increase interference among competing features and may encourage a greater reliance on higher-order regularities when distinguishing between similar alternatives [24]. Further converging evidence links healthy aging to reduced mnemonic precision and neural distinctiveness decline, both of which are associated with poorer mnemonic discrimination under interference and greater dependence on coarse, schema-like structures [43,52,72]. The present findings tie these threads together by suggesting that age differences are most evident when schematic expectations compete with fine-grained object identity and that this competition is reflected in converging behavioral biases and gaze dynamics. More broadly, the graded contrast between integrable and non-integrable schema deviation mirrors predictive processing accounts, wherein violation of expectation can enhance memory through informative prediction errors or hinder performance when prediction errors are too substantial to be accommodated within a given schema [33,48]. However, data from complementary learning systems frameworks are consistent with the idea that remembering schema violations requires balancing attention to item-specific traces with neocortical knowledge regulation, which may become more difficult with age [18,19,73].

Several limitations constrain the inferences that can be drawn from the present study. The cross-sectional design and the absence of direct assessments of sensory acuity and executive function capacity limit our ability to differentiate between perceptual and control-based explanations. Additionally, young adults approached the ceiling for schema-consistent objects, reducing sensitivity to detect condition differences. Alternative explanations therefore remain plausible, including age-related perceptual/oculomotor changes (e.g., reduced visual acuity/contrast sensitivity or oculomotor slowing), residual low-level visual differences (e.g., salience, visual complexity, or feature overlap), altered salience or weighting of schema deviations, and strategic shifts toward relying on schematic priors under uncertainty [71,74,75,76]. Although the RR–UR contrast was designed to isolate integrability, we cannot fully rule out that some portion of the effect reflects image-level differences. To mitigate this concern, we constructed stimuli as matched triads (NR/RR/UR) that shared the same cue and the diagnostic component within each set, holding primary perceptual overlap relatively constant while manipulating schematic deviation at the level of the completed object configuration. Moreover, retrieval choices were made among alternatives drawn from the same matched triad, reducing the likelihood that condition effects are driven by generic visual discriminability. Nevertheless, no stimulus set can eliminate all image-level variance; future work could therefore complement these design controls with computational estimates of salience and complexity (and related image-based metrics) to quantify and further constrain residual low-level differences [77,78,79]. While these constraints do not undermine the central pattern, they motivate targeted extensions combining richer individual difference measures with neural indices of control and hippocampal engagement during retrieval [80,81,82]. Such work could test whether gaze signatures that predict the successful rejection of schema-consistent lures in young adults are associated with frontal-hippocampal coordination and whether individual differences in reactive control capacity or interventions that bolster such processes are associated with reduced schema-consistent false memories in older adults [83].

In conclusion, the present study suggests that age-related differences in memory for schema-deviant objects are associated with decreased accuracy and a shift in the manner in which evidence is sampled and utilized during the retrieval process. Older adults exhibited an increase in schema-consistent false memory and a slower, less selectively adjusted allocation of attention when schema deviation was high. They also demonstrated a selective reduction in the degree to which gaze patterns tracked subsequent choices. By combining behavioral decomposition of false memories with eye tracking and representational similarity, this study advances beyond merely documenting age-related memory decline. Instead, it helps constrain candidate retrieval dynamics that may contribute to schema-deviant remembering.

## Data Availability

This study was pre-registered in the OSF (https://osf.io/k9e54?view_only=69663450425e48d68432ce533f17717d, accessed on 16 May 2025). De-identified data and scripts used in the manuscript can be viewed and downloaded from the OSF (https://osf.io/m5hz2/files/osfstorage, accessed on 24 January 2026).

## Supplementary Materials

The following supporting information can be downloaded at https://www.mdpi.com/article/10.3390/brainsci16030289/s1, Table S1. Number of valid eye movement data trials retained after quality control for both young and older adults; Table S2. Definition of key eye movement indicators and their relationship to memory; Table S3. Control analysis using valid trial counts as covariates for eye movement data analysis; Table S4. Comparison of *p*-values for eye movement metrics with and without FDR correction; Table S5. Results of the mixed-effects model at the trial level for eye movement metrics; Table S6. Results of RSA between young and older adults using Kendall’s tau and permutation test; Table S7. Results of RSA between young and older adults using bootstrap; Table S8. Key R packages and versions for data cleaning, statistical analyses, and visualization.
