# Supplementary material for: Aging Weakens Memory for Schema-Deviant Objects and Decouples Gaze Sampling from Retrieval Decisions
Source: Brain Sci. 2026 Mar 5;16(3):289. doi: 10.3390/brainsci16030289 (PMC13024725; doi:10.3390/brainsci16030289)
Supplement: Supplementary file 1 [file brainsci-16-00289-s001.zip › brainsci-4142192-supplementary.pdf]

Supplemental materials

Table S1. Number of valid eye movement data trials retained after quality control for both young and older adults.

Condition	YA		OA	
	M	*SD*	*M*	*SD*
NR	17.77	2.32	17.14	2.40
RR	17.77	1.90	16.00	3.54
UR	17.27	2.60	15.52	3.53

Note: YA, young adults; OA, older adults; NR, non-restructured; RR. reasonably restructured; UR, unreasonably restructured.

Table S2. Definition of key eye movement indicators and their relationship to memory.

Indicator	Operational definition	Relationship with memory
Target viewing proportion	In a single trial, the total fixation duration within the target AOI is divided by the sum of the total fixation durations across the three types of AOIs (NR, RR, and UR).	According to the "target-competition" framework, the extent to which attention resources are allocated to the target object reflects the advantage of target-related extraction evidence in competition.
Early target viewing proportion	After the trial begins, only use the first five fixations. Calculate the total fixation duration within the target AOI and standardize it using the total fixation duration across all three types of AOIs within the same time frame as the denominator.	To more closely capture early attentional orientation, one must consider whether target information can be prioritized for quicker processing during the initial retrieval stage.
First fixation latency	The time from the start of the trial until gaze first enters the target AOI, which is aligned to 0 at the onset of the first fixation in that trial.	This indicates the speed at which the target object is located or contacted, which may reflect the accessibility of target cues and the efficiency of retrieval guidance.
First fixation duration	This is the duration of the first fixation when the target	It may reflect the intensity with which the initial sampling and

	AOI is initially entered.	evaluation of evidence is conducted upon first contact with the target information. However, it could also be influenced by competition and uncertainty, in which case it would require interpretation alongside other indicators.
Mean fixation duration	This is the average duration of all fixations within the target VOI.	The sustained processing time scale on target information may be related to demands for evidence integration or decision load. This is not equivalent to a simple "good/bad" memory.
Revisit rate	Count the number of visits to the target AOI during the trial. Each entry into the target area from outside counts as one visit. Calculate the proportion of non-first visits. If the target area is never entered, record it as missing.	The tendency to sample back and forth between the target and competing information may reflect the need for monitoring and verification, as well as the inhibitory load, when competition intensifies.

Note: NR, non-restructured; RR, reasonably restructured; UR, unreasonably restructured; AOI, area of interest.

Table S3. Control analysis using valid trial counts as covariates for eye movement data analysis.

Indicators	Main effects				Interaction	
	Age		Condition			
	F	*p*	*F*	*p*	*F*	*p*
Target viewing proportion	3.85	0.051	49.38	<0.001	3.64	0.028
Early target viewing proportion	20.72	<0.001	59.11	<0.001	13.50	<0.001
First fixation latency	85.71	<0.001	66.96	<0.001	5.18	0.007
First fixation duration	1.22	0.274	40.06	<0.001	4.53	0.013
Mean fixation duration	7.63	0.008	28.51	<0.001	13.35	<0.001
Revisit rate	29.94	<0.001	35.36	<0.001	1.17	0.314

Table S4. Comparison of *p*-values for eye movement metrics with and without FDR correction.

Indicators	Main effects				Interaction	
	Age		Condition			
	p	p_{FDR}	p	p_{FDR}	p	p_{FDR}
Target viewing proportion	0.008	0.010	<0.001	<0.001	0.032	0.039
Early target viewing proportion	<0.001	<0.001	<0.001	<0.001	<0.001	<0.001
First fixation latency	<0.001	<0.001	<0.001	<0.001	0.015	0.022
First fixation duration	0.139	0.139	<0.001	<0.001	0.011	0.022
Mean fixation duration	0.005	0.007	<0.001	<0.001	<0.001	<0.001
Revisit rate	<0.001	<0.001	<0.001	<0.001	0.407	0.407

Table S5. Results of the mixed-effects model at the trial level for eye movement metrics.

Indicators	Main effects						Interaction		
	Age			Condition					
	F	*p*	p_{FDR}	*F*	*p*	p_{FDR}	*F*	*p*	p_{FDR}
Target viewing proportion	7.42	0.009	0.010	69.34	<0.001	<0.001	3.51	0.037	0.044
Early target viewing proportion	43.19	<0.001	<0.001	74.49	<0.001	<0.001	14.60	<0.001	<0.001
First fixation latency	126.55	<0.001	<0.001	154.51	<0.001	<0.001	10.19	<0.001	<0.001
First fixation duration	1.87	0.177	0.177	36.55	<0.001	<0.001	3.97	0.023	0.035
Mean fixation duration	8.26	0.006	0.009	22.42	<0.001	<0.001	12.31	<0.001	<0.001
Revisit rate	37.63	<0.001	<0.001	39.44	<0.001	<0.001	1.13	0.332	0.332

Table S6. Results of RSA between young and older adults using Kendall's tau and permutation test.

Indicators	YA (M±SD)	OA (M±SD)	*Mean differences*	$p_{permutation}$	p_{FDR}
Target viewing proportion	0.57±0.17	0.60±0.17	-0.04	0.442	0.663
Early target viewing proportion	0.52±0.18	0.36±0.20	0.15	0.004	0.009
First fixation latency	−0.13±0.18	−0.14±0.22	0.004	0.934	0.934
First fixation duration	0.40±0.20	0.25±0.19	0.15	0.005	0.009
Mean fixation duration	0.48±0.19	0.27±0.24	0.21	<0.001	0.004
Revisit rate	0.35±0.24	0.37±0.20	-0.01	0.809	0.934

Table S7. Results of RSA between young and older adults using bootstrap.

Indicators	*df*	*t*	*p*	p_{FDR}	*Cohen's d*	95% CI of *d*
Target viewing proportion	52.49	-1.39	0.171	0.256	-0.37	[–0.92, 0.21]
Early target viewing proportion	51.72	4.04	<0.001	0.001	1.08	[0.57, 1.71]
First fixation latency	52.67	0.95	0.345	0.413	0.26	[–0.28, 0.78]
First fixation duration	41.19	2.18	0.035	0.069	0.60	[0.13, 1.09]
Mean fixation duration	50.73	3.73	<0.001	0.001	1.01	[0.47, 1.64]
Revisit rate	52.98	0.26	0.792	0.792	0.07	[–0.44, 0.62]

Table S8. Key R packages and versions for data cleaning, statistical analyses, and visualization.

Category	Package	Version
Eye-tracking data import/processing	eyelinkReader	1.0.3
Data cleaning / wrangling	dplyr	1.1.4
Data cleaning / wrangling	tidyr	1.3.1
Data structures	tibble	3.2.1
Data import	readr	2.1.6
String processing	stringr	1.5.1
Functional programming	purrr	1.2.0
Statistical analysis (utility)	bruceR	2025.8
Statistical tests / summaries	rstatix	0.7.2
Data visualization	ggplot2	4.0.1
Figure composition	patchwork	1.3.2
Export / reporting	export	0.3.2
